# Management of Acute Infrainguinal Graft Occlusion: Surgical and Endovascular Approaches in Contemporary Practice

**DOI:** 10.3390/jpm15120568

**Published:** 2025-11-28

**Authors:** Edoardo Pasqui, Leonardo Pasquetti, Greta Ferraro, Bruno Gargiulo, Cecilia Molino, Elisa Lazzeri, Giuseppe Galzerano, Gianmarco de Donato

**Affiliations:** Vascular Surgery Unit, Department of Medicine, Surgery and Neuroscience, University of Siena, 53100 Siena, Italy

**Keywords:** acute limb ischemia, infrainguinal bypass, graft occlusion, thrombolytic therapy, thrombectomy, peripheral arterial disease, vascular surgical procedures

## Abstract

**Background**: Acute infrainguinal bypass graft occlusion is a critical vascular emergency that threatens limb viability and challenges both surgical and endovascular management. Despite progress in revascularization strategies, outcomes remain suboptimal, and consensus on the optimal treatment approach is lacking. **Methods**: This narrative review summarizes current evidence on the epidemiology, etiology, diagnosis, and treatment of acute infrainguinal graft occlusion. Particular attention is given to the evolving role of catheter-directed thrombolysis and mechanical thrombectomy, as well as to prevention strategies based on structured surveillance and medical optimization. **Results**: Infrainguinal bypass failure is influenced by technical, anatomical, and systemic factors, with distinct mechanisms affecting vein and prosthetic grafts. While surgical thrombectomy remains a viable option in selected cases, endovascular techniques have gained prominence due to their minimally invasive nature and promising short-term outcomes. Prevention of occlusion through duplex surveillance and best medical therapy is crucial to preserving graft patency and reducing major amputation risk. **Conclusions**: Management of acute graft occlusion requires timely diagnosis and a tailored, multidisciplinary approach. Although endovascular therapies have expanded treatment options, further prospective studies are needed to define optimal strategies and improve long-term outcomes in this high-risk population.

## 1. Introduction

Peripheral arterial disease (PAD) is a progressive atherosclerotic condition that affects more than 200 million individuals worldwide and represents a major cause of morbidity and mortality, particularly in elderly populations. Among its clinical manifestations, chronic limb-threatening ischemia (CLTI) is the most severe and debilitating, often requiring repeated surgical and endovascular revascularization to avoid limb loss. Infrainguinal bypass surgery remains a cornerstone of treatment in selected patients, especially when endovascular approaches are not feasible or have failed.

Despite advances in surgical techniques, anaesthetic management, and perioperative care, the durability of infrainguinal bypass remains a significant concern. Graft failure, especially when it occurs acutely, can lead to severe consequences such as recurrent ischemia, limb loss, and increased mortality. Acute graft occlusion is a vascular emergency that requires prompt diagnosis and appropriate therapeutic intervention. Sub-acute or chronic reocclusion, however, does not always constitute a vascular emergency but presents a genuine challenge for treatment. Nonetheless, its management continues to present clinical difficulties due to heterogeneous presentations, varying graft characteristics, and evolving endovascular and surgical strategies.

**Figure 1:**
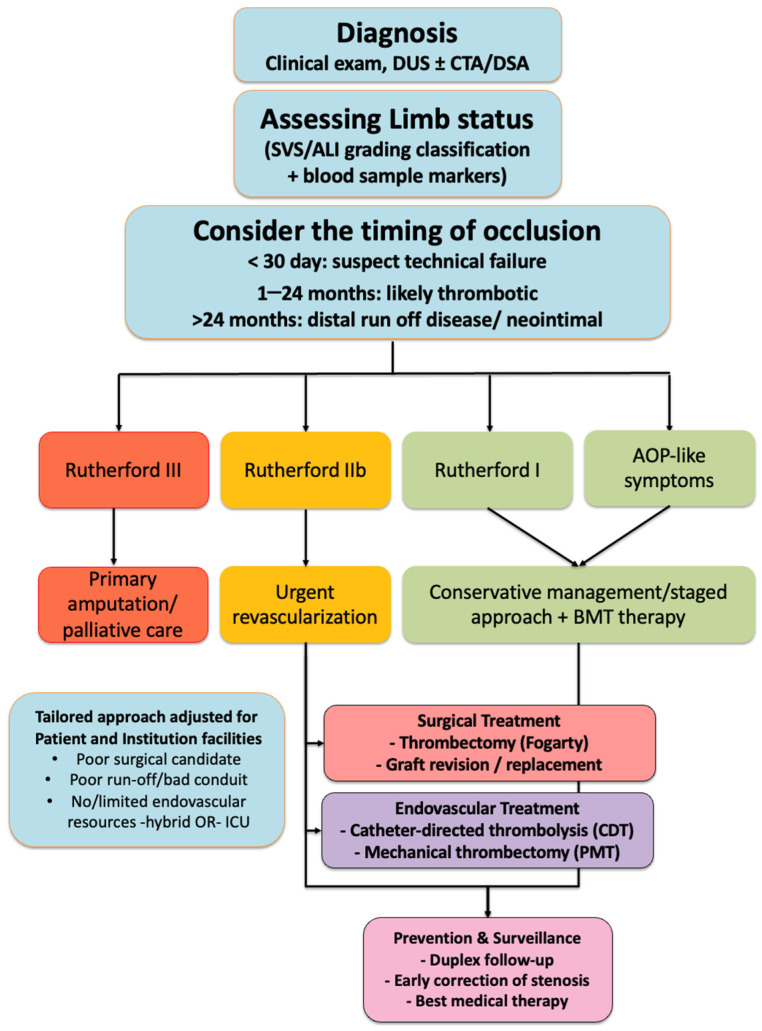
Flow-chart summarizing the management of acute infrainguinal graft occlusion. After diagnosis (clinical examination, Duplex Ultrasound (DUS) ± Computed Tomography Angiography (CTA)/Digital Subtraction Angiography (DSA)), limb viability is assessed according to the Society for Vascular Surgery (SVS) classification. Treatment options include conservative management with the best medical therapy in asymptomatic patients or those with good collaterals, surgical treatment (thrombectomy with a Fogarty catheter, graft revision, or replacement), and endovascular treatment (Catheter-Directed Thrombolysis (CDT), or Percutaneous Mechanical Thrombectomy (PMT)). Regardless of the chosen strategy, prevention and surveillance consist of duplex follow-up, early correction of stenosis, and best medical therapy.

This narrative review aims to provide a comprehensive overview of acute infrainguinal bypass graft occlusion, exploring its epidemiology, pathophysiology, clinical presentation, diagnostic work-up, and current treatment modalities, including surgical and endovascular techniques. Emphasis is placed on recent advances in catheter-directed thrombolysis and mechanical thrombectomy, as well as on the importance of prevention and postoperative surveillance (Figure 1).

## 2. Epidemiology

Infrainguinal bypass remains a widely adopted technique in the management of complex PAD and CLTI, particularly in patients with long-segment occlusions, heavily calcified vessels, or failed endovascular interventions. Autogenous great saphenous vein (GSV) grafts are preferred due to their superior patency, but prosthetic grafts (such as PTFE or Dacron) are still frequently used when vein conduits are unavailable or unsuitable.

The reported 1-year primary patency rate of infrainguinal bypass grafts varies widely in the literature, ranging from 40% to 88% [1], depending on the patient population, conduit type, target vessel, and technical factors. Vein grafts generally exhibit superior long-term performance compared to prosthetic conduits, particularly in below-the-knee locations [2].

Occlusion of a bypass graft may occur early (within 30 days), intermediate (1 month to 2 years), or late (>2 years), each with distinct underlying mechanisms and clinical implications [3]. While vein graft occlusion is most common in the early postoperative period due to technical issues or low-flow states, prosthetic grafts are associated with a sustained risk of occlusion that can persist for years, with late failure rates reaching up to 40% at 5 years [4].

The incidence of acute bypass occlusion is difficult to ascertain precisely due to variability in definitions and reporting standards. However, registry data and single-center series suggest that acute graft occlusion may account for 10% to 20% of all presentations of acute limb ischemia (ALI) [5]. When left untreated, acute bypass failure carries a high risk of major amputation and mortality, particularly in frail or comorbid patients. Many patients with infrainguinal bypass grafts do not present at the time of their first occlusion. It is not uncommon for presentation to occur after multiple graft failures, often during the third or even fourth episode, especially in individuals with advanced atherosclerosis or inadequate surveillance. These recurrent events reflect the chronic, relapsing nature of peripheral arterial disease and highlight the need for long-term follow-up and secondary prevention strategies.

Beyond the type of conduit and the timing of failure, several additional factors may predispose to graft occlusion. For example, spliced grafts or composite conduits, often used when the great saphenous vein is of insufficient length, tend to perform less reliably than uninterrupted vein segments. Poor quality vein (varicose lesions, vein with a thick wall, post-thrombotic consequences, vein diameter < 3 mm) represents a major concern for autologous graft durability [6]. Similarly, repeat or secondary bypass procedures, typically carried out in patients with failed prior revascularizations, are often more challenging due to scar tissue, limited landing zones, and diminished runoff quality. In these cases, the target vessels are frequently smaller and more diseased, further increasing the risk of early or late graft failure. On the other hand, prosthetic grafts (e.g., PTFE or Dacron) are associated with significantly higher rates of mid- and long-term failure, especially in below-the-knee positions where flow is typically low. Their thrombogenic surface, lack of endothelialization, and poor compliance mismatch with native vessels promote neointimal hyperplasia at anastomoses and chronic thrombotic occlusion. Furthermore, infection risk is greater with prosthetic materials, further compromising graft patency [7]. When the great saphenous vein is unavailable or unsuitable, several alternative conduits can be considered. Upper extremity veins, such as the cephalic or basilic veins, could be used and may offer acceptable patency. Spliced vein grafts or composite conduits (e.g., prosthetic with vein cuff) can be employed, though with potentially reduced durability. In infected or hostile surgical fields, cryopreserved allografts or biosynthetic grafts may be used as salvage options, albeit with variable long-term outcomes.

Poor distal runoff remains one of the most critical determinants of graft patency. Even the best surgical reconstruction may be compromised if the outflow bed, comprising tibial and pedal arteries, is inadequate. In such scenarios, slow flow and turbulent hemodynamics promote thrombus formation and reduce the effectiveness of the graft over time.

Patient-specific factors also play a crucial role. Conditions associated with a hypercoagulable state, such as active malignancy, antiphospholipid antibody syndrome, or other prothrombotic disorders, can predispose to early graft thrombosis despite technically sound procedures. Similarly, systemic atherosclerosis, diabetes, renal insufficiency, and poor cardiovascular reserve negatively affect vascular healing and long-term conduit performance. While data remain limited, some studies suggest that young patients may experience early graft failure due to undiagnosed hypercoagulable states or autoimmune disorders, rather than technical causes [8]. Obesity may also contribute to graft failure by increasing technical complexity, promoting a pro-thrombotic milieu, and reducing mobility and surveillance adherence. However, evidence remains inconclusive and larger studies are needed to establish definitive associations [9]. Racial disparities have been observed in peripheral arterial disease outcomes. For instance, African American patients experience higher rates of graft failure, limb loss, and reintervention, likely due to a combination of biological factors, socioeconomic barriers, and limited access to follow-up care [10]. While specific data on racial disparities in infrainguinal graft occlusion are sparse, this issue warrants further investigation and consideration in future clinical guidelines.

Ultimately, graft patency is determined by a complex interplay between surgical technique, conduit biology, anatomical characteristics, and systemic factors. While the surgeon’s expertise and choice of graft material are undoubtedly important, the broader clinical picture, including the patient’s comorbidities and the quality of the native vasculature, often exerts an equally decisive influence. For this reason, long-term graft surveillance and aggressive medical optimization are essential components of post-operative management, aiming to the risk of thrombotic events and extend the durability of sation.

## 3. Methods

This article was conceived as a narrative review with the aim of providing a clinically oriented and pragmatic synthesis of the available literature on the management of acute infrainguinal bypass graft occlusion. To ensure adequate coverage of relevant evidence, we performed a non-systematic literature search across three major databases: PubMed, Scopus, and the Cochrane Library. The search covered the period from January 2000 to April 2024, and was conducted using various combinations of keywords, including: infrainguinal bypass, bypass graft occlusion, acute limb ischemia, thrombolysis, mechanical thrombectomy, surgical revascularization, limb salvage, hybrid procedures, prosthetic graft, and vein graft.

We focused on original articles reporting clinical outcomes related to the treatment of acute graft occlusion, including randomized trials, prospective and retrospective studies, registry data, and large case series. Only English-language studies involving human subjects were considered. Editorials, expert opinions without original data, case reports, and studies unrelated to revascularization strategies were excluded.

Although we did not apply a formal quality assessment tool such as GRADE or ROBIS, we critically appraised the clinical relevance, methodological clarity, and applicability of each study included in our discussion. The objective was not to provide a systematic quantitative synthesis, but rather to construct a real-world, experience-based overview of surgical, endovascular, and hybrid strategies, integrating available data with current vascular practice.

Throughout this review, outcome data are reported according to the definitions used in the original studies. When specified, primary patency refers to uninterrupted graft function without any reintervention, whereas secondary patency indicates maintained graft function following reintervention. Technical success denotes immediate restoration of flow post-procedure, while clinical success reflects sustained limb perfusion and symptom resolution during follow-up. Where studies did not specify these definitions, this limitation has been acknowledged.

## 4. Diagnosis

Recognizing acute infrainguinal bypass graft occlusion promptly is essential, as timely diagnosis significantly influences the chances of limb salvage and overall patient outcomes. However, the clinical presentation can vary widely, depending on factors such as the timing of the occlusion, the type of graft used, the condition of the distal vasculature, and the degree of collateral circulation.

Typically, acute graft occlusion presents with a sudden onset of ischemic symptoms. Patients may report intense rest pain, limb coldness, or paresthesia. In more severe cases, signs such as motor weakness or paralysis may indicate an advanced stage of ALI. The classic clinical picture may include the so-called “five Ps”: pain, pallor, pulselessness, paresthesia, and paralysis, though not all signs are always present. In contrast, chronic graft failure tends to develop more insidiously, with symptoms such as worsening claudication, new-onset rest pain, or the appearance of non-healing ulcers or tissue loss, hallmarks of CLTI [11].

Interestingly, not all patients with graft occlusion exhibit overt ischemia. In some cases, well-developed collateral circulation may mitigate symptoms, resulting in a more indolent course or even asymptomatic presentation. On the other hand, the occluded graft itself may become a source of thromboembolic material, leading to peripheral embolization and a more complex clinical scenario [12].

The clinical assessment remains the cornerstone of diagnosis. Physical examination may reveal a cold, pale extremity with delayed capillary refill and absent distal pulses. In patients with a known history of infrainguinal bypass, any acute deterioration in walking distance, rest pain, or visible skin changes should immediately raise suspicion for graft failure. Palpation of the graft, when superficial, may suggest thrombosis if pulsatility is lost.

To classify the severity of ischemia, the Society for Vascular Surgery (SVS) has developed a widely used clinical staging system that distinguishes between viable, threatened, and irreversible limbs. This classification helps determine whether urgent intervention is needed and can guide the choice between surgical and endovascular options. Similarly, the SVS WIfI classification, taking into account the severity of wounds, ischemia, and foot infection, is valuable in stratifying the risk of amputation and guiding treatment in the context of chronic or borderline presentations [13]. In this perspective, patients with Rutherford IIb ischemia, characterized by motor deficits and severe pain, require emergent surgical revascularization, ideally within 1 h to avoid irreversible tissue loss. In contrast, those with Rutherford IIa ischemia, showing only sensory deficits or mild rest pain, may be candidates for a more nuanced approach, including endovascular-first strategies such as catheter-directed thrombolysis or mechanical thrombectomy, provided that institutional expertise and resources are available.

Laboratory investigations may support the diagnostic work-up and guide treatment decisions. D-dimer levels may be elevated in thrombotic events, although they lack specificity. Serum creatinine is essential to assess the safety of contrast-based imaging and to monitor renal function, especially in the setting of thrombolysis or extensive ischemia. A complete blood count and coagulation profile should be obtained, particularly in candidates for thrombolysis. Inflammatory markers such as C-reactive protein (CRP) and white blood cell count may help detect underlying graft infection, especially in the presence of systemic symptoms or fever.

In cases of prolonged or severe ischemia, evaluation of muscle cytonecrosis markers such as creatine kinase, lactate dehydrogenase, and serum or urinary myoglobin is recommended to assess the extent of tissue injury and the risk of reperfusion syndrome. Elevated levels may also inform the need for intensive care monitoring, early fasciotomy, or renal protective strategies [14]. The diagnostic pathway often begins with bedside tools such as a handheld Doppler, which can provide immediate information about the presence or absence of flow in the graft and distal arteries. A sudden loss of Doppler signal in a previously patent bypass is highly suggestive of thrombosis. Non-invasive vascular imaging, particularly duplex ultrasonography (DUS), is typically the first-line diagnostic modality. DUS can confirm graft occlusion, localize the thrombus, and assess the quality of distal runoff. While highly informative, its accuracy may be limited in certain situations, such as in obese patients or when heavy arterial calcification is present. In some cases, acute worsening of limb perfusion may not be solely attributable to graft occlusion. Reduced cardiac output due to acute heart failure or myocardial infarction can diminish collateral circulation, mimicking or exacerbating peripheral ischemia. A comprehensive cardiovascular assessment is essential in patients with borderline findings or unclear symptoms to avoid unnecessary interventions and to identify systemic contributors to impaired perfusion.

To better define the anatomy and plan appropriate intervention, cross-sectional imaging is often required. Computed tomography angiography (CTA) is commonly used for this purpose, as it offers high-resolution images of the entire vascular tree, including the graft, anastomoses, and distal arteries. CTA is fast, widely available, and useful for preoperative planning. Magnetic resonance angiography (MRA), although less frequently employed in the acute setting, may be preferred in patients with contraindications to iodinated contrast media.

Digital Subtraction Angiography (DSA) remains the gold standard for imaging acute infrainguinal graft occlusions, providing excellent spatial resolution and allowing for direct intervention. Historically, DSA posed concerns in patients with renal dysfunction or iodinated contrast allergy. However, the use of carbon dioxide (CO_2_) angiography has significantly reduced these limitations, making DSA feasible in a broader range of patients. Nevertheless, DSA may still be limited in the presence of heavy calcifications or low-flow runoff, which can impair contrast distribution. Additionally, CO_2_ angiography is not universally available, and operator experience is crucial to ensure optimal image quality and safety [15]. While DSA can generally detect both large and small thrombotic occlusions, very distal lesions (e.g., tibial or pedal vessels) may be underestimated without magnified or oblique projections. In some cases, adjunctive imaging such as intravascular ultrasound (IVUS) [16] may improve lesion visualization and procedural planning. Moreover, diffuse thrombotic occlusion may lead to underestimation of lesion length, which can affect treatment strategy.

In summary, the diagnosis of acute infrainguinal graft occlusion requires a high index of suspicion, especially in patients with a history of prior bypass surgery and sudden changes in limb symptoms. A structured and timely diagnostic work-up, beginning with clinical evaluation and bedside Doppler, followed by targeted imaging, is essential to guide effective intervention and improve patient outcomes.

## 5. Guidelines and Best Medical Therapy

The management of acute infrainguinal bypass graft occlusion poses a considerable clinical challenge, and current international guidelines offer only limited direction on how to approach this scenario. Most recommendations focus more broadly on the treatment of ALI or the prevention of graft failure, rather than addressing acute graft occlusion as a distinct clinical entity. As such, decision-making often relies heavily on clinician experience, institutional protocols, and the specific clinical context.

European guidelines, for instance, discuss graft occlusion mainly within the framework of ALI, recommending prompt identification and treatment of the underlying cause [17]. While they do not provide a dedicated algorithm for acute bypass thrombosis, they emphasize the importance of selecting patients appropriately for initial surgery, employing meticulous surgical technique, and maintaining close postoperative surveillance as key strategies to prevent graft failure. In particular, the use of autogenous great saphenous vein grafts remains strongly recommended due to their superior long-term patency [18,19]. The importance of a structured follow-up program, incorporating regular imaging and clinical evaluation, is also consistently emphasized across major vascular societies.

Given the lack of high-level evidence and the heterogeneity of available studies, therapeutic decisions in the acute setting must be individualized. In some patients, particularly those with minimal or no ischemic symptoms and well-compensated collateral circulation, a conservative approach may be appropriate. This is especially true in high-risk surgical candidates or in cases where the limb is not immediately threatened. However, such an approach requires careful monitoring, as the natural history of untreated graft thrombosis is associated with poor outcomes.

Conservative management of acute graft occlusion has been linked to significantly poorer outcomes compared to revascularization strategies. In a descriptive analysis by Feliz and colleagues, the one-year rate of major amputation or death following first-time infrainguinal bypass occlusion was nearly 60% in patients who did not undergo reintervention [20]. This sharply contrasts with lower rates observed in patients who received graft salvage procedures or underwent repeat bypass surgery. These findings strongly support the idea that, when possible, active intervention offers the best chance of preserving limb function and improving survival.

Nonetheless, best medical therapy (BMT) plays a fundamental role in both the immediate and long-term management of patients with bypass grafts. BMT encompasses antithrombotic and/or anticoagulant therapy, lipid-lowering agents, blood pressure control, and glycemic optimization [21]. Its role extends beyond the prevention of graft failure to reducing cardiovascular risk and improving overall outcomes in patients with peripheral arterial disease. Unfortunately, real-world data have shown significant variability in the prescription and implementation of these therapies, highlighting the need for better adherence to evidence-based protocols.

In the context of graft occlusion, medical therapy may serve as an adjunct to revascularization or as a primary strategy in selected patients. In cases where immediate intervention is not pursued, close clinical follow-up combined with optimized BMT may help stabilize the condition and delay or prevent progression to critical ischemia. In addition, ongoing surveillance with duplex ultrasound and clinical assessments is essential to detect changes that might necessitate a shift in management strategy [22].

In summary, while guidelines provide a general framework, the management of acute graft occlusion remains highly individualized. Conservative management may be reasonable in selected asymptomatic cases, but most patients benefit from prompt revascularization. Regardless of the initial approach, BMT is indispensable and should be optimized in all patients with infrainguinal bypass grafts to support both graft patency and overall vascular health.

## 6. Surgical Treatment

Surgical revascularization has historically represented the first-line approach in the management of acute infrainguinal bypass graft occlusion. Before the advent and widespread adoption of endovascular therapies, open surgical techniques were the mainstay of treatment for thrombosed grafts, particularly in patients with threatened limbs. The fundamental principle of surgical management is the prompt removal of thrombotic material, restoration of graft patency, and correction of any underlying technical or anatomical defect that may have contributed to the occlusion.

One of the earliest and most widely used techniques involved the use of a Fogarty balloon catheter to perform surgical thrombectomy [23]. This approach typically includes exposure of the graft, passage of the catheter through the occluded segment, and extraction of thrombus, followed by careful inspection of the anastomotic sites. When performed promptly and successfully, this technique can restore flow through the graft and salvage the threatened limb.

However, despite its simplicity and initial effectiveness, surgical thrombectomy is often insufficient as a standalone solution. Several studies and clinical experiences have highlighted the limitations of this approach. Residual thrombus in the distal runoff vessels is a frequent finding and may go undetected during surgery, especially if intraoperative imaging is not employed. Moreover, thrombus adherent to the native arterial wall, particularly in the proximal inflow tract, may resist mechanical extraction and compromise the overall result [24].

To address these limitations, intraoperative completion angiography has become a valuable adjunct. Providing real-time visualization of the arterial tree, it allows the surgeon to assess the adequacy of thrombus removal, identify any residual stenosis or embolic debris, and, when necessary, proceed with additional interventions [25]. In some cases, a hybrid approach combining open thrombectomy with intraoperative endovascular treatment, such as balloon angioplasty or stenting of residual lesions, may be the most effective strategy. Reoperation may also involve graft revision or complete replacement. In the presence of graft degeneration, anastomotic aneurysm, infection, or technical failure, replacing the graft with a new conduit, preferably an autologous vein, if available, may offer better long-term results. In many cases, aggressive arterial reconstruction to all levels of the leg, combined with follow-up and reintervention, when necessary, can salvage up to 86% of critically threatened limbs at intermediate follow-up, as demonstrated by Cheshire et al. [26]. However, redo bypass surgery is technically more demanding and carries higher perioperative risks, particularly in elderly or comorbid patients. Scar tissue, altered anatomy, and limited availability of suitable target vessels all contribute to the complexity of the procedure.

Despite these challenges, surgical treatment continues to play a crucial role in selected cases. It remains a preferred option in patients with contraindications to thrombolytic therapy, such as recent major surgery, intracranial pathology, or active bleeding. Moreover, in limbs with advanced ischemia, where time is critical and rapid flow restoration is needed, open surgery may provide the fastest and most effective solution.

Nevertheless, the long-term outcomes of surgical revascularization for acute graft occlusion are variable. Studies have reported relatively high rates of re-occlusion and major amputation, especially in the presence of poor distal runoff or advanced comorbid conditions. These observations have fueled growing interest in less invasive strategies that may offer comparable efficacy with reduced procedural morbidity. In patients with severely compromised distal runoff, poor cardiac reserve, or multiple prior revascularizations, further attempts at graft salvage may carry limited benefit and significant risk. In such scenarios, the option of primary major amputation should be openly discussed with the patient and caregivers. This approach, while often perceived as a last resort, may offer better outcomes in terms of pain control, mobility with prosthesis, and quality of life compared to repeated high-risk procedures.

While surgical treatment was once the standard of care for acute bypass occlusion, it is now often reserved for specific clinical situations where endovascular options are contraindicated, unavailable, or have failed. Careful patient selection, intraoperative imaging, and hybrid approaches are key to maximising the success of surgical revascularization in this complex and high-risk population. The management of graft failure also depends on the timing of the occlusion. Early failure (within 30 days) is typically related to technical issues or hypercoagulable states and often requires surgical revision or thrombectomy. Intermediate failure (1 to 24 months) may be addressed with endovascular reintervention, mechanical thrombectomy, or redo bypass, depending on the anatomical and clinical context. Late failure (after 2 years) is often driven by neointimal hyperplasia or disease progression and can be managed with a combination of endovascular and surgical techniques, tailored to the patient’s anatomy and comorbidities.

## 7. Endovascular Treatment

Over the past two decades, endovascular therapy has emerged as a central component in the management of acute infrainguinal bypass graft occlusion. Initially developed as an alternative for patients unfit for surgery or with high anaesthetic risk, endovascular techniques have progressively gained favour due to their minimally invasive nature, shorter recovery times, and growing body of supporting evidence. Among these, catheter-directed thrombolysis (CDT) and percutaneous mechanical thrombectomy (PMT) have taken on a prominent role, often used individually or in combination, depending on the clinical context.

## 8. Catheter-Directed Thrombolysis

CDT was one of the first endovascular options introduced for ALI and has been extensively studied since the 1990s. In this technique, a thrombolytic agent, typically urokinase or alteplase, is infused directly into the thrombus through a multi-sidehole catheter positioned within the occluded segment. By delivering the drug locally and continuously, CDT facilitates gradual clot dissolution and can restore graft patency while minimising systemic exposure and reducing the risk of bleeding.

Thrombolysis is particularly well-suited to infrainguinal bypass occlusion because it offers the possibility of treating not only the occluded graft itself but also the native vessels distal to the graft, which are frequently involved in the thrombotic process. Importantly, thrombolysis can expose the underlying cause of the occlusion, such as an anastomotic stenosis or a distal lesion, thus allowing for subsequent definitive treatment with angioplasty or stenting.

Numerous studies have examined the outcomes of CDT in this setting. For example, Conrad and colleagues reported a 71% success rate in re-establishing graft patency in a cohort of 69 occluded infrainguinal bypasses (48 vein grafts and 21 prosthetic grafts) [27]. They emphasized the importance of combining thrombolysis with correction of the underlying lesion to optimize long-term results. Other studies have found comparable or even superior patency rates compared to surgery, particularly when thrombolysis is followed by targeted endovascular interventions. For example, in 2009, a prospective nonrandomized controlled study reported the results of a 56 cohort of patients with occluded bypasses. Primary rates of 72.9% at 1 year after surgery and 77.9% at 1 year after CDT with or without major adjunctive surgery were outlined. Amputation-free survival was quite similar between the two groups (86.4% in CDT group vs. 87.5% in surgery group) [28]. In 2016, Schrijver et al. reported their experience with CDT for acute lower extremity occlusions involving both native arteries and prosthetic bypass grafts. In the subgroup analysis focusing on bypass grafts, complete lysis (>95%) was achieved in 69% of cases. Major hemorrhagic complications occurred in 7% of patients, while the 30-day mortality and amputation rates were 1% and 13%, respectively. Over a mean follow-up period of 27 ± 19 months, amputation-free survival was 78% at 1 year and decreased to 51% at 5 years. Despite encouraging early technical success, the authors concluded that long-term outcomes remained suboptimal, with a relatively low amputation-free survival over time [29].

In a more recent study, although not specifically limited to acute presentations, Betz et al. reported that 78.8% of occluded grafts were successfully reopened using intra-arterial thrombolysis. Notably, in 36.6% of cases, thrombolysis alone was sufficient to restore patency without adjunctive procedures. Thrombolysis-related complications were observed in 15.4% of patients [30].

However, thrombolysis is not without limitations. The process may take several hours to achieve full clot resolution, and during this period, the limb remains at risk if perfusion is severely compromised. Furthermore, systemic absorption of thrombolytic agents can lead to bleeding complications, which range from minor hematomas to life-threatening hemorrhages. Although rare, severe events such as intracranial bleeding underscore the need for careful patient selection and close monitoring during therapy [31].

Recent innovations such as ultrasound-accelerated thrombolysis have sought to enhance the efficacy and safety of CDT. By using ultrasonic energy to disrupt fibrin structure and facilitate drug penetration, these systems have demonstrated reduced infusion times and lower required doses, although robust evidence from large trials remains limited.

## 9. Mechanical Thrombectomy

To address the limitations of thrombolytic therapy, percutaneous mechanical thrombectomy has been introduced as a complementary or alternative strategy. PMT devices allow for the physical removal of thrombus from the graft lumen, often without the need for prolonged lytic infusion. As a result, they are especially useful in patients at high risk of bleeding or in those with contraindications to thrombolysis.

Several mechanical thrombectomy systems have been evaluated in the setting of bypass graft occlusion. Among them, the Rotarex^®^ system has demonstrated promising results, with reported technical success rates approaching 100% in small series. This device uses a rotating helix within a catheter to fragment and aspirate thrombus, enabling rapid flow restoration. In one study, Lichtenberg and colleagues treated 22 patients with acute femoropopliteal bypass occlusion, both venous and prosthetic, achieving full revascularization in nearly all cases, with minimal need for adjunctive thrombolysis [32]. In addition, Heller et al. identified a total of 147 ALI patients who underwent mechanical thrombectomy using Rotarex system for the treatment of ALI in infrainguinal occlusions. Notably, 27 patients (18%) presented with bypass occlusion. Although a subgroup analysis was not conducted, outcomes are promising. They achieved 90.5% procedural revascularization success rate when combining mechanical thrombectomy with limited thrombolysis for severe outflow obstruction, with only 1 death and 3 amputations. The primary success was achieved in 68.7% of the patients with the mechanical thrombectomy only, and in 21.8% of the patients, they successfully used additional limited thrombolysis in the outflow. The overall mortality was 0.7% and the amputation rate was 2% at 30 days [33].

Other devices, such as the AngioJet™ rheolytic thrombectomy system (Boston Scientific, Marlborough, MA, USA) and the Indigo™ aspiration system, have also shown favourable outcomes. These systems use either high-pressure saline jets or continuous aspiration to remove thrombus and have been associated with high rates of technical success and limb salvage, even in complex or delayed presentations. In particular, Steinberger et al. evaluated rheolytic mechanical thrombectomy for the treatment of ALI secondary to thrombosis in infrainguinal PTFE bypass grafts in 83 patients. The procedural success was achieved in 60/83 (72%) cases, procedure failure was seen in 11/83 (13%) cases, and CDT was administered in 68/95 (72%) of patients. At mean follow-up (10 months), amputation-free survival was 80%, 20% of patients had undergone amputation, and 12% underwent delayed revision. Mortality at follow-up was 13% [34]. In the INDIAN trial, patients with ALI were treated with first-line mechanical thrombectomy using the Indigo Aspiration System (Penumbra, Alameda, CA, USA), prior to any adjunctive therapy such as angioplasty, stenting, or surgery, according to physician’s discretion. The occlusions involved native arteries in 68% of cases (102 out of 150 patients), while 32% (48 patients) had ALI secondary to prior peripheral endovascular or open surgical intervention. Specifically, graft occlusion was identified in 16 patients, including 14 prosthetic and 2 autologous vein grafts. Following the Indigo procedure, all patients demonstrated a thrombo-aspiration in peripheral ischemia (Thrombo-aspiration In Peripheral Ischemia, TIPI) score of 2–3, indicating effective revascularization. Overall, primary technical success (TIPI 2–3 flow) was achieved in 88.7% of patients, while assisted primary technical success increased to 95.3% (143 out of 150 patients) after adjunctive interventions [35]. These encouraging results were confirmed in the subsequent INDIAN UP trial. In this cohort, primary technical success was achieved in 90.8% of patients, and assisted primary technical success reached 96.4%. Among these, 26 patients had prior peripheral bypass grafts, 21 prosthetic and 5 using the autologous great saphenous vein [36].

The STRIDE study, an international, multicenter, prospective trial, further evaluated the Indigo system in 119 patients with lower extremity ALI. While outcomes were generally favourable, the utility of the device in bypass graft occlusion remains less well defined. Twenty-seven patients in the study had a bypass in the target leg, but the exact location of the thrombus was not specified, and patients with saphenous vein graft thrombosis were excluded. Moreover, one of the two major amputations reported occurred after repeated occlusion of a prosthetic bypass, highlighting the potential complexity of these cases [37].

**Figure 2:**
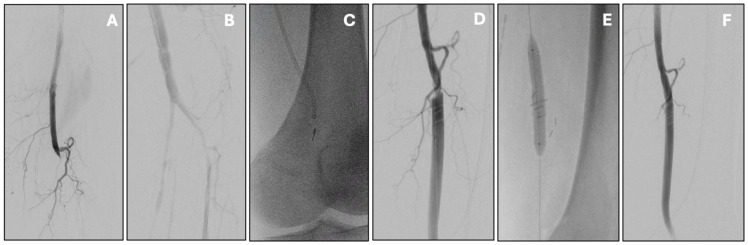
Acute infrainguinal bypass occlusion managed by percutaneous mechanical thrombectomy with the Indigo Penumbra system. Initial angiography showed popliteal graft thrombosis (**A**) with distal re-opacification of the popliteal artery (**B**). Mechanical thrombectomy was carried out with multiple passes of the Indigo Penumbra system (**C**), resulting in effective thrombus removal. This also uncovered a proximal anastomotic stenosis (**D**), which was subsequently treated with stenting (**E**). Final angiography confirmed good patency of the treated segment without residual stenosis or thrombus (**F**).

The Indigo Aspiration System functions by delivering continuous aspiration through a high-efficiency catheter, allowing for clot removal in a single-pass or multiple-pass technique. The system is designed to be flexible and trackable in torturous anatomy, making it suitable for peripheral interventions. Recent generations of the Indigo system have integrated artificial intelligence–driven clot detection and flow optimization algorithms. These innovations enable real-time adjustment of suction power and catheter positioning, potentially improving procedural efficiency and reducing time to revascularization. (Figure 2).

While data on these newer models are still emerging, preliminary reports suggest enhanced clot clearance and reduced need for adjunctive therapies.

Despite these advances, the evidence base for PMT remains somewhat limited, particularly in comparison to more established techniques. Most published data come from retrospective series or single-center experiences, often involving small numbers of patients. Furthermore, while the short-term results are encouraging, long-term patency and limb salvage rates remain to be fully defined.

Nevertheless, mechanical thrombectomy offers a valuable option in patients with severe ischemia, especially when used in combination with low-dose thrombolytics or as part of a hybrid strategy. Its ability to rapidly restore perfusion, reduce hospital stays, and avoid systemic lysis makes it an increasingly attractive tool in the vascular specialist’s armamentarium.

In conclusion, endovascular therapies have become a cornerstone in the treatment of acute bypass graft occlusion. While thrombolysis remains a well-established and widely used approach, mechanical thrombectomy offers an appealing alternative, particularly in high-risk or time-sensitive scenarios. In clinical practice, the choice between these techniques often depends on patient characteristics, anatomical considerations, available resources, and operator expertise. Indeed, not all centers, even in high-income countries, have access to repetitive angiography, ICU-level monitoring, or CO_2_ angiography during prolonged thrombolytic infusions. In such settings, mechanical thrombectomy or hybrid approaches may be preferred over catheter-directed thrombolysis to ensure timely and effective revascularization. Tailoring treatment based on local capabilities remains a key component of patient-centered care.

## 10. Prevention and Surveillance

Preventing graft failure, particularly acute occlusion, remains one of the most challenging aspects of post-revascularization care. Despite successful surgical or endovascular procedures, the durability of infrainguinal bypass grafts is constantly threatened by restenosis, thrombosis, and disease progression. Early detection of these complications, while the graft is still patent, provides the best opportunity to intervene and preserve limb function.

Stenosis, in particular, is a well-recognized precursor to thrombosis. It may develop in up to 40% of infrainguinal bypass grafts, often at anastomotic sites or within the vein conduit itself [38]. This process is driven by intimal hyperplasia, altered hemodynamics, and ongoing atherosclerotic disease. Once a graft becomes occluded, treatment becomes more complex, the risk of limb loss increases, and the likelihood of achieving long-term patency diminishes. For this reason, the cornerstone of prevention is vigilant postoperative surveillance.

DUS is the most effective and widely adopted tool for graft surveillance. It offers a non-invasive, reproducible, and cost-effective means of assessing graft patency, detecting stenotic lesions, and evaluating flow characteristics in both the conduit and the native vessels proximal and distal to the graft. Regular surveillance with DUS during the first year after surgery is especially critical, as this is the period when the risk of stenosis is highest. Studies have shown that timely detection of hemodynamically significant lesions followed by minor corrective procedures, such as angioplasty or revision, can significantly prolong graft longevity and reduce the risk of occlusion [39,40].

In addition to imaging, clinical evaluation remains an essential component of surveillance. Any change in walking capacity, return of rest pain, or new onset of tissue loss should prompt immediate reassessment. Even subtle signs, such as decreased skin temperature or delayed capillary refill, may indicate compromised flow and warrant further investigation.

An interesting area of ongoing discussion concerns the role of supervised exercise therapy, particularly structured walking programs, as a component of conservative management in selected patients with graft occlusion. While most evidence supporting walking therapy derives from studies on chronic peripheral arterial disease and intermittent claudication, some principles may also apply to patients with non-critical bypass thrombosis [41]. Walking-induced ischemia stimulates angiogenesis and collateral vessel recruitment, potentially improving perfusion in compromised limbs. In patients who do not present with critical limb ischemia and have stable symptoms, walking therapy, combined with close clinical and imaging surveillance, may serve as a temporising or stabilising strategy, especially in those at high operative risk. However, data specific to graft thrombosis are lacking, and this approach should be reserved for carefully selected cases with preserved limb viability and frequent reassessment.

Equally important in the prevention of graft failure is the optimization of BMT [42].

Antithrombotic therapy plays a pivotal role in the long-term success of infrainguinal bypass grafts. While single antiplatelet therapy (usually aspirin) remains the cornerstone of secondary prevention, recent evidence supports the addition of low-dose anticoagulation in selected high-risk patients. The VOYAGER PAD trial demonstrated that rivaroxaban 2.5 mg twice daily, when added to aspirin, reduced the risk of acute limb ischemia, major amputation, and cardiovascular events in patients undergoing lower limb revascularization. Although the trial included both surgical and endovascular patients, subgroup analyses suggested benefit regardless of revascularization type [43,44].

Nonetheless, robust data specifically addressing graft patency in bypass patients are still lacking, and bleeding risk must be carefully considered, especially in elderly or frail individuals. In patients with a history of graft thrombosis, hypercoagulable states, or recurrent occlusions, a more aggressive antithrombotic regimen (e.g., anticoagulation with or without antiplatelet therapy) may be considered. Shared decision-making and close clinical surveillance remain essential.

Lipid-lowering therapy, blood pressure control, smoking cessation, and tight glycemic management are also critical to minimising disease progression. Despite their proven efficacy, these measures are often underutilized or inconsistently applied in clinical practice. Real-world studies have documented significant variability in the prescription and adherence to BMT, suggesting that opportunities for secondary prevention are frequently missed [45,46].

Furthermore, patient education and structured follow-up protocols can play a decisive role in long-term success [47]. Ensuring that patients understand the importance of medication adherence, routine monitoring, and early reporting of symptoms is vital. Equally, standardized surveillance programs, tailored to the type of graft, anatomical site, and patient comorbidities, can help identify issues early and allow for timely re-intervention before irreversible damage occurs.

**Figure 3:**
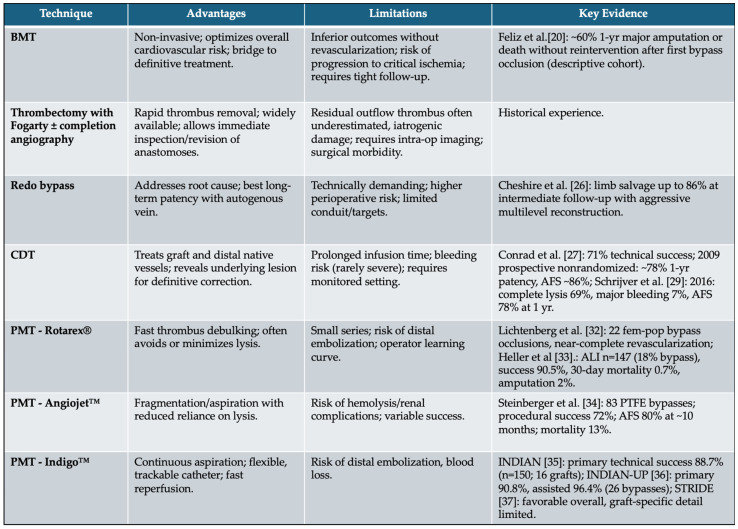
Full spectrum of conservative and surgical strategies applicable to acute infrainguinal bypass occlusion. Advantages, limitations and current key evidence are shown. (BMT: best medical therapy, CDT: catheter-direct thrombolysis, PMT: percutaneous mechanical thrombectomy).

In summary, preventing acute infrainguinal graft occlusion requires a proactive, multi-faceted approach that combines clinical vigilance, structured imaging surveillance, and meticulous medical management. While surgical and endovascular techniques are essential tools in the treatment of graft failure, it is the long-term follow-up strategy that ultimately determines the durability and success of the revascularization. The following table, represents a sum-up of all the treatment possibilities (Figure 3).

## 11. Conclusions

Acute occlusion of an infrainguinal bypass graft represents one of the most feared and complex complications in peripheral arterial surgery. Despite significant improvements in surgical techniques, conduit selection, and perioperative care, graft failure remains a substantial cause of morbidity, limb loss, and mortality, particularly in patients with advanced peripheral arterial disease and chronic limb-threatening ischemia.

Timely recognition of acute graft occlusion is essential, as delayed diagnosis can lead to irreversible ischemic damage and loss of revascularization opportunity. While physical examination and duplex ultrasound remain the mainstays of initial assessment, cross-sectional imaging and angiography are often necessary to define the extent of occlusion and plan intervention.

Before selecting the optimal treatment strategy, a structured assessment of patient risk and procedural feasibility is essential. The decision-making process should begin with a careful evaluation of patient-related factors such as age, comorbidities, and overall functional status, which influence both procedural tolerance and long-term prognosis.

Equally important are graft-specific features, including the type of conduit (autologous vein versus prosthetic), the anatomic location, and the time elapsed since graft creation, as these elements often determine whether failure is of a technical, thrombotic, or progressive nature.

The degree of limb threat, typically graded according to the Rutherford classification, remains the cornerstone for immediate triage: limbs with motor deficit or sensory loss require urgent surgical revascularization, whereas milder ischemia may allow for a more selective, endovascular-first approach.

Finally, treatment selection must account for institutional resources and technical expertise. Availability of hybrid operating rooms, endovascular devices, and experienced teams often dictates whether a surgical, endovascular, or hybrid strategy can be performed safely and effectively.

Management strategies have evolved substantially over the past two decades. Surgical thrombectomy and graft revision, once considered the standard of care, still play an important role in selected cases, particularly when rapid flow restoration is required or when thrombolytic therapy is contraindicated. However, the advent of catheter-directed thrombolysis and percutaneous mechanical thrombectomy has transformed the treatment landscape, offering less invasive alternatives with favorable short- and mid-term outcomes. These techniques not only allow for thrombus clearance but also enable the identification and treatment of underlying lesions, with the potential to prolong graft patency and delay the need for repeat surgery.

Nonetheless, acute treatment alone is not sufficient to ensure long-term success. Prevention of graft thrombosis must remain a primary objective. Structured surveillance with duplex ultrasound, early detection of stenosis, and prompt reintervention are critical to maintaining patency. At the same time, the optimization of the best medical therapy, including antithrombotic and lipid-lowering regimens, must be universally adopted and standardized across care settings.

Despite the clinical relevance of acute bypass graft occlusion, the current evidence base remains limited and fragmented. Most available studies are retrospective, single-center series with small sample sizes, heterogeneous populations, and non-standardized outcome definitions. Comparative data between surgical and endovascular approaches are scarce, and randomized controlled trials are lacking. In addition, long-term follow-up data, patient-reported outcomes, and cost-effectiveness analyses are rarely reported.

There is a pressing need for prospective, multicenter studies and real-world registries to better understand treatment outcomes, refine decision-making algorithms, and evaluate emerging technologies. Future research should also focus on the integration of functional outcomes, quality of life metrics, and the role of personalized antithrombotic strategies in optimizing long-term success and patient satisfaction. In the meantime, clinicians must continue to rely on a patient-centered, multidisciplinary approach that integrates technical expertise, careful follow-up, and rigorous medical management. Only through such comprehensive care can we hope to improve outcomes in this challenging and high-risk population.

## Data Availability

No new data were created or analyzed in this study. Data sharing is not applicable to this article.

## Abbreviations

The following abbreviations are used in this manuscript:

PAD	Peripheral Arterial Disease
CLTI	Chronic Limb-Threatening Ischemia
GSV	Great Saphenous Vein
SVS	Society for Vascular Surgery
DUS	Duplex UltraSonography
CTA	Computed Tomography Angiography
MRA	Magnetic Resonance Angiography
DSA	Digital Subtraction Angiography
BMT	Best Medical Therapy
CDT	Catheter-Directed Thrombolysis
PMT	Percutaneous Mechanical Thrombectomy
ALI	Acute Limb Ischemia
TIPI	Thrombo-Aspiration in Peripheral Ischemia
